# Protocol for microfluidic-based high-precision general polarization fluorescence microscopy of lipid packing in membrane vesicles

**DOI:** 10.1016/j.xpro.2026.104525

**Published:** 2026-04-27

**Authors:** Gaukhar Zhurgenbayeva, Nika Otrin, Chee Seng Man, Christian Eggeling, Ziliang Zhao

**Affiliations:** 1 Institute of Applied Optics and Biophysics, Friedrich-Schiller-Universität Jena, Jena, Germany; 2 Leibniz Institute of Photonic Technologies, Member of the Leibniz Centre for Photonics in Infection Research (LPI), Jena, Germany; 3 Process Systems Engineering, Max Planck Institute for Dynamics of Complex Technical Systems, Sandtorstrasse 1, 39106 Magdeburg, Germany; 4 Max Planck School Matter to Life, Germany; 5 Jena Center for Soft Matter (JCSM), Philosophenweg 7, 07743 Jena, Germany

**Keywords:** Biophysics, Cell Membrane, Microscopy

## Abstract

Lipid packing plays a critical role in membrane organization and function, but its precise quantification remains challenging in fluidic environments. Here, we present a protocol to integrate confocal fluorescence generalized polarization (GP) imaging with a polydimethylsiloxane (PDMS)-based microfluidic platform. We describe steps for lipid preparation, giant unilamellar vesicle (GUV) fabrication, microfluidic trapping, and GP analysis using polarity-sensitive dyes. This protocol enables robust measurement of membrane order and is readily adaptable to diverse lipid compositions and aqueous environments.

For complete details on the use and execution of this protocol, please refer to Zhao et al.^1^^,^^2^

## Highlights

• Steps for microfluidic device fabrication and assembly
• Procedures for giant unilamellar vesicle (GUV) production and entrapment
• Instructions for confocal general polarization (GP) imaging and lipid packing assessment

## Abstract (editor highlights)

Publisher’s note: Undertaking any experimental protocol requires adherence to local institutional guidelines for laboratory safety and ethics.

## Graphical abstract

**Figure:**
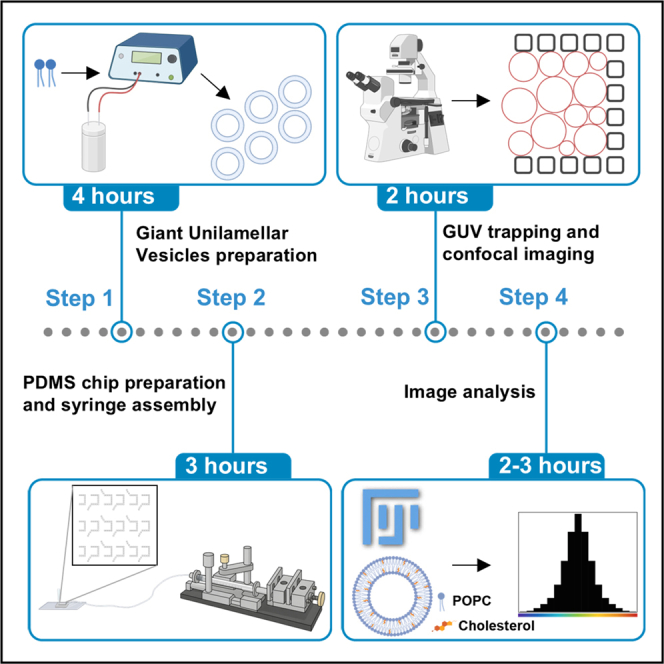

## Before you begin

Membrane lipid packing is one of the key determinants of membrane organization. Fluorescence-based generalized polarization (GP) microscopy employing polarity-sensitive dyes provides a sensitive measure of lipid packing, but achieving precise quantification is often hindered by sample drift of e.g., membrane vesicles such as giant unilamellar vesicles (GUVs) in aqueous environments.

Conventional ways to tackle the issue of sample drifts involve utilizing either a sticky surface from a dried thin layer of bovine serum albumin (BSA) or streptavidin-biotin affinities to immobilize GUVs onto the coverslip surface. Nevertheless, these endeavors only result in wobbly GUVs, and could potentially alter local membrane compositions, induce shape transformations, as well as eventually lead to inaccurate analysis on lipid packing.^3^^,^^4^^,^^5^ To address these limitations, this protocol utilizes polydimethylsiloxane (PDMS)-based microfluidic chips to immobilize GUVs without perturbing their integrity. This setup enables stable two-channel confocal GP imaging of GUV membranes by employing polarity-sensitive solvatochromic dyes such as Nile Red derivatives like NR12A or classical Laurdan probes. These dyes’ emission spectra shifts in response to changes in membrane lipid packing order, exhibiting a red shift in less packed and thus more disordered membrane environments and a blue shift in more ordered environments.^6^ Such environment-sensitive probes provide robust optical readouts of membrane biophysical properties like lipid packing and order,^7^ membrane viscosity,^8^ and membrane tension,^9^ which are all inter-connected.

Using these environment-sensitive probes, membrane lipid packing or order is commonly quantified using the GP parameter, which is usually calculated from intensities collected in two spectral channels, one at a shorter more blueish and one at a longer more reddish wavelength-range. Specifically, *I*_*o*_ (ordered) and *I*_*d*_ (disordered) refer to the fluorescence intensities measured from the shorter and longer wavelength regions of the emission spectrum,^10^^,^^11^ respectively.

(Equation 1) $$GP=\frac{\left(I_{o}-I_{d}\right)}{\left(I_{o}+I_{d}\right)}$$

Higher GP values indicate more ordered (tightly packed) lipid phases, whereas lower values correspond to more disordered (loosely packed) membranes. The GP readout thus requires fluorescence detection in two distinct wavelength ranges, which can be achieved, for example, using two detectors equipped with defined bandpass filters or by employing spectral detection.^12^ The GP readout should not be confused with fluorescence polarization readout, which relies on physical polarizers to measure fluorescence intensities at defined polarization orientations.

Here, we describe the specific procedures for comparing lipid packing between two different lipid compositions in GUVs. Although the protocol utilizes a special Nemesys S syringe pump and Abberior Infinity Line for showcasing the GUV trapping and imaging parameters, it is important to note that the recommended settings are universal and could be easily realized with setups from other brands. This approach also readily accommodates GUVs with bending rigidities from soft, fluid-phase membranes to more rigid compositions, and is also robustly generalizable for assessing the impact of various factors on the membrane lipid order, such as the influence by membrane-interacting agents like toxins, peptides, viruses, or drugs. Furthermore, our GP measurements can also be straightforwardly performed on a super-resolution STED microscope to achieve higher spatial resolution on membrane lipid packing analysis.^13^ In addition, with appropriately adjusted emission filter sets, the method can be extended to other solvatochromic dyes (e.g., Laurdan and its structural derivatives).

The main advancement in our approach is the microfluidic-assisted trapping, which relies on hydrodynamic force balancing and channel confinement to immobilize GUVs, providing stable positioning, minimal perturbation and continuous environmental control by maintaining the imaging target in the focal spot of the confocal fluorescence microscope for an accurate and non-biased assessment of GP values, which makes it particularly suitable for high-precision fluorescence imaging experiments. The following describes necessary preparations beforehand.

### Innovation

The innovation of this experimental configuration lies in the integration of the microfluidic platform for the trapping and immobilization of GUVs for high precision GP imaging. Conventional studies of GUVs are often constrained due to the free-floating vesicles, which introduce challenges such as positional drift and limited image acquisition times. The use of a microfluidic chip addresses these limitations by enabling stable immobilization of individual vesicles without the need for chemical fixation or lipid alteration, thereby preserving the structural and biophysical integrity of the vesicles. This immobilization strategy ensures reproducible positioning and long-term observation of GUVs, which significantly enhances the spatial and temporal resolution of GP imaging. Furthermore, the combination of controlled microfluidic confinement with a lipid-order–sensitive imaging modality provides a unique means to quantitatively probe membrane phase behavior, heterogeneity, and dynamic responses under well-defined experimental conditions. The approach also offers scalability, as the microfluidic design allows parallel immobilization of a large population of GUVs, facilitating robust systematic and statistically accurate analysis, bringing ultimate efficiency for comparative studies. Thus, the synergistic coupling of microfluidic trapping and GP imaging ensures a powerful platform for high-resolution and high-throughput investigation of membrane biophysical properties.

### Microfluidic wafer design and fabrication

**Timing: 1 day**

1. Design the microfluidic wafer master.
  a. Create the wafer master layout using AutoCAD.
    i. Draw an 8-channel cascade trapping system, with each channel containing 17 GUV traps.
    ii. Draw guiding posts inside each channel to divert flow toward the traps.
    iii. Set the gap between guiding posts to 10 μm to ensure efficient GUV capture.

**Figure 1:**
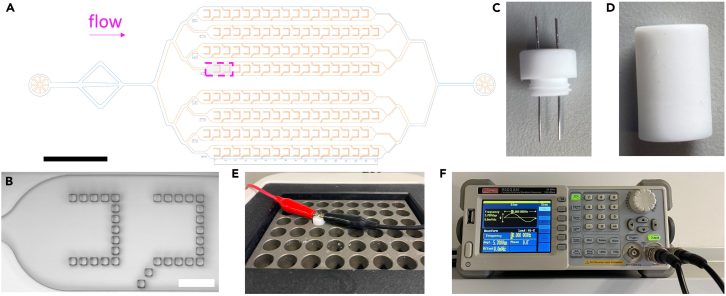
Overview of the microfluidic chip design and electroformation setup for GUV preparation (A) Structure layout of the microfluidic device. Scale bar: 2 mm. (B) Confocal image illustrating the detailed architecture of the trap corresponding to the magenta-highlighted region in (A). Scale bar: 200 μm. Figures 1A and 1B are adapted from Zhao et al.^1^ under the Creative Commons CC BY license. (C) PTFE chamber cap integrated with Pt wires for AC field application. (D) PTFE chamber equipped with threading for secure cap fixation. (E) Fully assembled chamber showing the closed cap and electrical connections via alligator clips for AC field application. (F) Function generator displaying the output parameters used for GUV fabrication.

***Note:*** Refer to Figures 1A and 1B for schematic representations and detailed chip geometry.

2. Prepare the silicon wafer for photoresist coating.
  a. Place a 4-inch silicon wafer in an oven at 150°C for 10 min to dehydrate surface.

***Note:*** Dehydration enhances SU-8 adhesion and prevents delamination during development.

3. Apply SU-8 photoresist to the wafer.
  a. Pour SU 8-3050 photoresist (Kayaku Advanced Materials) onto the dehydrated wafer.
  b. Spin-coat the photoresist at the rotation speed required to obtain a 40 μm thickness.
  c. Pre-bake the coated wafer in following steps:
    i. 65°C for 1 min.
    ii. 95°C for 13 min.
    iii. 65°C for 1 min.
4. Write the patterned design onto the wafer with UV exposure.
  a. Place the pre-baked wafer into a mask aligner (UV-KUB 3, KLOE).
  b. Align the photomask designed in AutoCAD with the wafer.
  c. Expose the wafer for 7.1 s at a total dose of 250 mJ/cm^2^.
5. Post-bake the UV-exposed wafer.
  a. Immediately after exposure, bake the wafer at:
    i. 65°C for 1 min.
    ii. 95°C for 4 min.
    iii. 65°C for 1 min.
6. Develop the wafer.
  a. Immerse the wafer in SU-8 developer (Kayaku Advanced Materials) to dissolve the excessive portion of photoresist on the wafer.
  b. Rinse the wafer with isopropanol.
  c. Dry the wafer gently with nitrogen gas.
  d. Hard-bake the wafer at 150°C.
7. Silanize the wafer to extend the lifespan and facilitate PDMS peeling.
  a. Place the hard-baked wafer in a glass Petri dish.
  b. Prepare several small aluminum foil cups.
  c. Add a few drops of trimethylchlorosilane (TMCS) into each cup.
  d. Distribute filled cups around the wafer without direct contact.
  e. Close the Petri dish with a lid and incubate for 12–16 h.

**Caution:** Handle TMCS exclusively inside a fume hood; the reagent is corrosive and moisture reactive.

### Prepare software and macro

**Timing: 30 min**

8. Install Fiji.
  a. Navigate to https://fiji.sc.
  b. Download the appropriate version of Fiji for your operating system.
  c. Install Fiji following the on-screen instructions.

***Note:*** Fiji is required for image preprocessing and downstream analysis.

9. Install the Fluidity Calculator plugin.
  a. Go to the GitHub repository at: https://github.com/pcarravilla/fluiditycalculator/tree/main.
  b. Download the plugin files as instructed in the repository.
  c. Install the plugin into Fiji by following the provided installation steps.

***Note:*** Ensure that the plugin loads without errors by restarting Fiji and checking the Plugins menu.

### Calibrate the osmometer

**Timing: 0.5–1 h**

10. Prepare osmometer for calibration.
  a. Turn on the Gonotec® Osmomat 3000 and allow it to equilibrate.
  b. Clean the sampling probe by wiping it thoroughly with a Kimtech™ wipe moistened with Milli-Q water.

**CRITICAL:** Proper probe cleaning prevents cross-contamination and ensures accurate osmolality readings.

11. Perform calibration using standard solutions from Gonotec GmbH.
  a. Load the 0 mOsmol/kg Milli-Q water onto the probe and start the measurement.
  b. Remove the sample and clean the probe.
  c. Measure the 300 mOsmol/kg and 850 mOsmol/kg calibration standard solutions.

**CRITICAL:** Always clean the probe between samples as in step 10 to avoid contamination.

12. Confirm that all readings fall within the acceptable tolerance range provided by the manufacturer.

## Key resources table

**Table:** 

REAGENT or RESOURCE	SOURCE	IDENTIFIER
**Chemicals, peptides, and recombinant proteins**		

MemGlow™ NR12A Membrane Polarity Probe	Cytoskeleton, Inc.	MG07
1-palmitoyl-2-oleoyl-glycero-3-phosphocholine, or 16:0-18:1 PC (POPC)	Avanti Polar Lipids	850457C
Cholesterol (ovine)	Avanti Polar Lipids	700000P
Trimethylchlorosilane (TMCS)	Merck	386529
Chloroform	Merck	132950
Sucrose	Merck	S0389
Glucose	Merck	G8270
Dimethyl sulphoxide (DMSO)	Carl Roth	A994.1
Bovine Serum Albumin (BSA)	Merck	A7906
Ethyl alcohol, pure	Merck	1.07017
Ethanol 70 %	Carl Roth	T913.5
2-Propanol	Merck	I9516
Sodium dodecyl sulfate (SDS)	Merck	L6026
Photoresist (SU-8 3050)	Kayaku Advanced Materials	Y311075
Developer	micro resist technology GmbH	mr-Dev 600
SYLGARD® 184 Silicone Elastomer Kit	Merck	761036
Calibration standard 300 mOsmol/kg NaCl/H_2_O	GONOTEC	30.9.0020
Calibration standard 850 mOsmol/kg NaCl/H_2_O	GONOTEC	30.9.0850

**Software and algorithms**		

AutoCAD	Autodesk	Version: S.51.0.0 AutoCAD 2022
CETONI Elements	Cetoni	https://cetoni.com/cetoni-elements/
Fiji	Open-source	RRID: SCR_002285
Fluidity Calculator plug-in	Carravilla et al., 2025^14^	https://github.com/pcarravilla/fluiditycalculator
Imspector Image Acquisition & Analysis Software	Abberior Instruments	N/A

**Other**		

Centrifuge 5804	Eppendorf	5804000013
Spin coater	Laurell	WS-650MZ-23NPPB
Baking oven	Binder	ED 23
Mask aligner	Kloe	UV-KUB 3
Hot plate	Torrey Pines Scientific	HP30A
Silicon wafer	Si-Mat	4″ Std Wafer, P/Bor
Chrome mask	Micro Lithography Services	N/A
Plasma Cleaner	Diener electronic	Zepto One
Waveform Generator	RS Components	123–6567
Ultrapure water system, Barnstead™ GenPure™	Thermo Scientific	BARN50131952
Ultrasonic cleaner	witeg Labortechnik	WUC-D03H
Precision Balance Pioneer™ Precision	OHAUS Europe	PA413
Vacuum pumping unit	Vacuubrand	MZ 2C NT
Vacuum desiccator	DWK Life Sciences	13485489
Osmomat Freezing Point Osmometer	GONOTEC	Model 3000 Basic
Industrial MultiMeter	EXTECH	EX503
Shaking Incubator, INCU-Line®	VWR	444–0732
Nemesys S syringe pump	Cetoni	NEM-B124-02 A
Dry Bath Heating block	witeg Labortechnik	DH.WHB00348
Infinity Line STED microscope	Abberior Instruments	N/A
1.0 mm biopsy punch	Kai Europe	49101
1.5 mm biopsy punch	Kai Europe	49115
MICROMAN™ Positive-Displacement Pipette 1–10 μL	Gilson	FD10001
MICROMAN™ Positive-Displacement Pipette 10–100 μL	Gilson	FD10004
MICROMAN™ Positive-Displacement Pipette 100–1000 μL	Gilson	FD10006
Capillary Pistons, TIPACK, 1–10 μL	Gilson	F148312
Capillary Pistons, TIPACK, 10–100 μL	Gilson	F148314
Capillary Pistons, TIPACK, 100–1000 μL	Gilson	F148560
Glass coverslips, 26 × 76 mm #1.5	Epredia	BC02600760AC40MNZ0
Gastight Instrument Syringe, 1 mL	Hamilton	81320
Bohlender™ PTFE tubing	Merck	Z609706
Metal connector	custom built	N/A
Upchurch PEEK Luer Adapter	Analytics Shop.com	UPP-659
Upchurch PEEK Fingertight Fitting	Analytics Shop.com	UPF-120x
Amber glass vials, 2 mL	Merck	27083-U
Petri dish (120 x 20 mm)	Carl Roth	KKA0.1
Aluminium foil	Toppits	N/A
Screw cap with PTFE liner	Merck	27091-U
Scalpel	Carl Roth	T997.1
Safe-Lock Tubes, 2 mL	Eppendorf	0030120094
Falcon 15 mL Conical Centrifuge Tubes	Fisher Scientific	11507411
Eppendorf Research Plus Pipette, 100–1000 μL	Eppendorf	3123000063
Eppendorf Research Plus Pipette, 20–200 μL	Eppendorf	3123000055
Pipette tips, 2–200 μL	BRAND	732008
Pipette tips, 50–1000 μL	BRAND	732012
Parafilm sealing film	Merck	HS234526B
PTFE sealing tape	Merck	Z104388
Scotch Magic invisible tape	3M	M8102566

## Materials and equipment

### Preparation of solutions

• Sucrose solution (S1).

To prepare a 300 mM sucrose solution, dissolve 513.4 mg of sucrose crystals in 5 mL of Milli-Q water.

***Note:*** Freshly prepared before use and store at 21°C–23°C for a maximum of 24 h.

• Isotonic sucrose–glucose solution (S2).

**Table:** 

Reagent	Final concentration	Amount
Sucrose	280 mM	479.2 mg
Glucose	20 mM	18.0 mg
Milli-Q water	N/A	5 mL

***Note:*** Freshly prepared before use and store at 21°C–23°C for a maximum of 24 h.

• BSA solution.

To prepare 10 mg/mL BSA solution, dissolve 100 mg of BSA powder in 10 mL of Milli-Q water. BSA solution can form foam, allow the solution to settle before further usage.

***Note:*** Freshly prepared before use and store at 4°C for a maximum of 7 days.

## Step-by-step method details

### Lipid mixture preparation for GUV formation

**Timing: 30 min**

The following steps describe the preparation of GUVs with defined lipid composition using dissolved lipid stock solutions. Two representative lipid mixtures are used as examples: pure POPC and POPC/cholesterol at a 70 : 30 molar ratio. The procedures can be further generalized to other lipid compositions.

1. Prepare lipid stock solutions in glass vials:
  a. Rinse the vials thoroughly with Milli-Q water, pure ethanol, followed by chloroform.
  b. Blow dry them with filtered compressed air between each washing step.
  c. Dissolve cholesterol powder in chloroform to a final concentration of 25 mg/mL.
  d. POPC is obtained pre-dissolved in chloroform at 25 mg/mL and can be used directly.

***Note:*** Store the lipid stock solutions in amber glass vials with screw caps at −20°C under inert gas (argon or nitrogen) to prevent lipid oxidation. Seal the vial caps first with PTFE sealing tape then parafilm, and store in light-protected containers.

**Caution:** Standard laboratory pipettes are not compatible with organic solvents such as chloroform. Use solvent-resistant pipettes and tips specifically designed for volatile chemicals.

2. Prepare lipid mixtures for different experimental conditions in chloroform (total volume 1 mL) from the above-mentioned stock solutions:
  a. Clean the vials as described in Step 1a and 1b above.
  b. Prepare pure POPC lipid solution (4 mM or 3.04 mg/mL), later referred to as L1:
    i. Pipette 121.6 μL of POPC stock solution, then add chloroform to reach a total volume of 1 mL.
  c. Prepare POPC: Cholesterol (POPC: Chol) mixture in 70 : 30 molar ratio (3.86 mM or 2.5 mg/mL), later referred to as L2:
    i. Pipette 82 μL of POPC stock solution and 18 μL of cholesterol stock solution, then add chloroform to reach a total volume of 1 mL.

### Electroformation and labeling of GUVs

**Timing: 3–4 h**

The following steps describe electroformation of GUVs using a homemade polytetrafluoroethylene (PTFE) chamber with a Platinum (Pt) wired cap (Figures 1C and 1D). An alternating electric field applied across the Pt electrodes facilitates swelling of thin lipid films into vesicles and subsequent vesicle detachment. Afterwards, we describe how to label electroformed GUVs with the solvatochromic membrane dye NR12A to enable fluorescence imaging.

3. Clean electroformation chamber.
  a. Immerse the two separated PTFE chamber parts in chloroform and sonicate for 5 min.
  b. Blow-dry the parts to remove residual chloroform with filtered compressed air.
  c. Rinse thoroughly with Milli-Q water.
  d. Blow-dry again with filtered compressed air.
4. Apply lipid mixtures.
  a. Spread 2–3 μL of lipid mixture evenly onto each of the two Pt wires.
5. Dry lipid films.
  a. Evaporate remaining chloroform from the lipid-coated wires by blowing Argon gas (∼10 s per wire).
  b. Place the cap with wires in a vacuum desiccator and dry the lipid films under vacuum for 1 h.
6. Add hydration solution.
  a. Fill the PTFE chamber with 400 μL of S1.
  b. Seal the chamber securely with the cap.
7. Apply electric field.
  a. Connect the function generator cables to the Pt wires using insulated alligator clamps (Figure 1E).
  b. Switch on the function generator.
  c. Apply a sinusoidal alternating current (AC) electric field at 10 Hz, 5.7 V peak-to-peak (Vpp) for 2 h at 21°C–23°C (Figure 1F).

***Note:*** The AC electric field can promote lipid film swelling and vesicle formation on the Pt wires.

***Note:*** Confirm the AC voltage at the Pt wires using a multimeter; the voltage across Pt wires should be ∼2 Vrms under these settings. Avoid direct contact between the wires or cables to prevent short circuits.

**CRITICAL:** For L2 mixtures, increase the electroformation time to at least 3 h and set the temperature of the heating block to 45°C to improve GUV size and yield.

8. Detach GUVs from Pt wires.
  a. Reduce the AC frequency to 2 Hz (maintain the same Vpp) and continue applying the AC field for an additional 30 min.

**Caution:** Chloroform is volatile and toxic. Work under a fume hood and dispose of chloroform waste in designated halogenated waste containers.

9. Collect GUVs.
  a. Turn off the function generator and disconnect the cables carefully.
  b. Unscrew the cap and collect the GUV-containing sucrose solution using pipette with a truncated 1000 μL pipette tip.
  c. Transfer the GUV suspension to a 2 mL Eppendorf tube.
  d. Clean the PTFE chamber immediately after use:
    i. Rinse with 2 % SDS solution followed by Milli-Q water.
    ii. Blow-dry with filtered compressed air.
    iii. Rinse with isopropanol and blow-dry again to remove solvents completely.
    iv. Store the chamber in air until next use.

**CRITICAL:** To prevent damage to the GUV membranes during collection, it is important to use truncated pipette tips to harvest them from the PTFE chamber to the 2 mL tube. Use a 1000 μL pipette tip and cut∼3–5 mm from the tip end with scissors. Standard tips generate high flow velocities and shear stress that can potentially rupture GUVs or lead to selective loss of larger ones.

10. Label GUVs.
  a. Prepare dye stock solution:
    i. Resuspend 4 nmol of lyophilized NR12A dye in 200 μL anhydrous DMSO to obtain a 20 μM stock solution.
      **CRITICAL:** Aliquot the dye solution in small volumes (e.g. 10–20 μL) and store at **−**20°C in dark to prevent photobleaching. Avoid repeated freeze-thaw cycles of the dye to maintain its stability.
  b. Transfer 200 μL of the GUV suspension to a 2 mL tube.
  c. Add 1 μL of 20 μM NR12A dye solution to the GUV suspension to reach a final dye concentration of 100 nM.
  d. Mix gently by tapping on the tube and incubate in dark at 21°C–23°C for 5 min.
    **CRITICAL:** Use the smallest possible volume of the dye when it is dissolved in DMSO, as excessive DMSO can disrupt lipid bilayers.
    **CRITICAL:** Check GUV size and yield on a glass coverslip using bright-field microscopy. This can be achieved by allowing the GUVs to sediment via the density gradient established by S1 and S2 across the GUV membrane (Refer to troubleshooting problem 2).

### Microfluidic chip preparation and syringe assembly

**Timing: 5 h**

The following steps describe the preparation and bonding of PDMS microfluidic chips to glass coverslips, followed by assembly of reservoirs, BSA coating and syringe pump setup.

**Figure 2:**
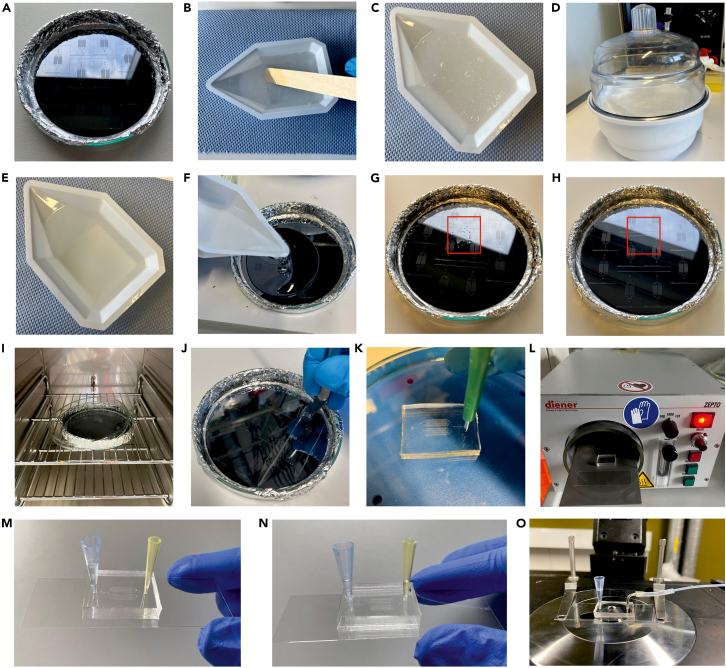
Fabrication and assembly process of the microfluidic chip (A) Wafer mounted in a glass Petri dish covered with aluminum foil. (B–E) Mixing and degassing of PDMS prior to mold casting. (F–H) Casting and degassing of PDMS on the patterned mold. (I–K) Curing, cutting and punching of the PDMS mold to form access ports. (L) Surface activation of the coverslip and PDMS block by plasma treatment prior to covalent bonding. (M) Oblique view of the microfluidic chip prior to BSA filling, where the aqueous level could only be visualized in the left blue tip. (N) Oblique view of the microfluidic chip following BSA filling, illustrating equal aqueous levels in both tips. (O) Bonded PDMS chip on glass substrate with inlet reservoir and outlet tubing attached, mounted on the microscope stage.
12. Mix the PDMS.
  a. Combine 10 parts silicone elastomer base with 1 part curing agent by mass in a plastic weighing bowl (e.g., 70 g PDMS + 7 g curing agent).
  b. Mix thoroughly using a wooden stick until the mixture becomes homogeneous (Figure 2B).
13. Degas and cast the PDMS.
  a. Degas the mixture in a vacuum desiccator to remove air bubbles (Figures 2C–2E).
  b. Pour the PDMS slowly onto the wafer to minimize bubble formation (Figure 2F).
  c. If bubbles remain near the design features, degas again until the surface is clear (see red square areas in Figures 2G and 2H).

***Note:*** Inspect the PDMS for dust or other particles and remove them with a needle if needed.

11. Prepare the wafer.
  a. Line a glass Petri dish with aluminum foil and place the wafer inside (Figure 2A).

14. Cure the PDMS block.
  a. Partially cover the Petri dish with its lid and place it in an oven at 80°C for 1 h (Figure 2I).
  b. Allow the PDMS to cool to 21°C–23°C afterward.
15. Cut the PDMS chip.
  a. Cut the cured PDMS into individual blocks using a scalpel (Figure 2J).

***Note:*** Avoid injury and protect the wafer surface while cutting.

16. Prepare the fluidic access ports.
  a. Place the PDMS chip with the microchannels side facing upward.
  b. Punch the inlet and outlet ports using a biopsy punch (Figure 2K).

***Note:*** The choice of biopsy punch size depends on the design of the microfluidic chip, as well as the type of tubing and connectors used. In our chip design, the inlet has a diameter of 1.5 mm and the outlet has a diameter of 1.0 mm.

**CRITICAL:** To prevent contamination of the flow channels by dust or other particles that could block the fluid flow, cover the channel surface with invisible tape (e.g., Magic™ Tape).

17. Clean glass coverslips for PDMS bonding.
  a. Clean both sides of glass coverslip with detergent for ∼20 s.
  b. Rinse the coverslip sequentially with Milli-Q water, 70% ethanol, then Milli-Q water again.
  c. Blow-dry with filtered compressed air.

***Alternatives:*** Soak coverslip in 2 % SDS solution for at least 2 h, then rinse and blow-dry.

***Note:*** Use filtered compressed air to remove any dust particles prior to bonding.

18. Activate surfaces using plasma cleaning.
  a. Place the chip and coverslip in a plasma cleaner with the microchannel side facing up (Figure 2L).
  b. Treat both surfaces with plasma for 60 s at ∼1 mbar.
19. Bond the PDMS chip to the coverslip.
  a. Align and place the PDMS block (microchannel side down) onto the coverslip immediately after plasma treatment.
  b. Observe the two surfaces coming into close contact as the air gap releases.
  c. Press the chip carefully to remove any visible air bubbles and ensure full surface contact.

***Note:*** Visually inspect the bonding process, pay special attention to critical structures such as posts or traps.

***Note:*** Place PDMS chip on the coverslip gently – do not apply force. The bonding process should be observable: when the chip is not in full contact with the glass, air bubbles are visible. Applying a gentle manual pressure, the air front should spread and eventually disappear.

20. Place the assembled chip in an oven at 80°C for 1 h to accelerate the chemical reaction of bonding.

***Alternatives:*** Leave the assembled microfluidic chip at 21°C–23°C for 12–16 h.

21. Assemble the PDMS chip.
  a. Prepare inlet and outlet reservoirs.
    i. Cut 200 μL and 1000 μL pipette tips to ∼2 cm in length.
    ii. Insert the 1000 μL tip into the 1.5 mm inlet and the 200 μL tip into the 1.0 mm outlet until both tips touch the coverslip base.

***Note:*** Adjust reservoir height to fit the microscope stage clearance.

22. Coat microfluidic channels with BSA.
  a. Add 50 μL of BSA solution to the inlet reservoir (Figure 2M).
  b. Place the chip in a centrifuge with appropriate holders and balance the setup with another chip.
  c. Centrifuge at 900 × *g* for 10 min.
  d. Confirm that the BSA solution equilibrates in both reservoirs post-spin (Figure 2N).

**CRITICAL:** Load BSA into the inlet in one continuous push to prevent air bubble introduction, as air bubbles introduced from the inlet can disrupt flow stability, and in some cases cause irreversible damage to the microfluidic device.

***Note:*** The holder inserts in the centrifuge should be either buckets or plate holders, depending on the centrifuge type.

23. Assemble the syringe.
  a. Rinse a 1 mL gastight Hamilton syringe with isopropanol and Milli-Q water, then blow-dry it.
  b. Assemble and pre-fill the syringe with S2.
    i. Start by first filling with 0.2 mL of S2 to eliminate bubbles, then continue filling up to 1 mL.

***Note:*** Bubble-free loading ensures consistent flow and prevents GUV deformation caused by flow instability induced by air bubbles.

24. Connect tubing to the syringe using a PEEK-luer adapter and fitting.

***Note:*** The tubing should be pre-assembled with a luer connector at the syringe end and a homemade metal connector at the chip end and stored in this way for daily usage.

25. Fill the tubing with S2 until it reaches the end of the metal connector.
26. Continue filling to a total volume of 0.8 mL.

***Note:*** This copious rinsing ensures bubble-free liquid inside the tubing.

27. Leave 0.2 mL of S2 in the syringe.
28. Remove the outlet reservoir gently.
29. Insert the metal tubing connector into the outlet port until it touches the coverslip.

**CRITICAL:** Ensure a small droplet is present at the end of the metal connector to maintain a smooth water-to-water interface and prevent air bubble introduction.

30. Transfer the assembled chip onto the microscope stage for GUV trapping (Figure 2O).
31. Set up the syringe pump.
  a. Turn on the Cetoni Base pump.
  b. Launch the Cetoni Elements software, the main control panel will appear (Figure 3A).

**Figure 3:**
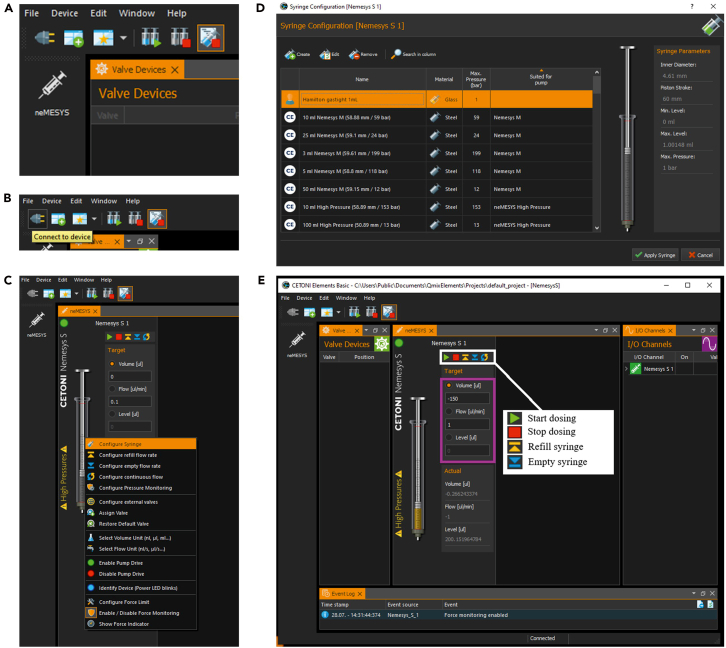
Operation of the Cetoni syringe pump using Cetoni Software for GUV trapping (A) Top left corner of the main operation window of the Cetoni software. (B) “Connect to device” button highlighted in yellow as indicated by the plug icon. (C and D) Steps for configuring the correct syringe type. (E) Final setup screenshot displaying the selected syringe type along with control buttons including “Start dosing”, “Stop dosing”, “Refill syringe” and “Empty syringe” (highlighted in white box) used for operating the syringe pump during the trapping experiment.
  c. Click “Connect to device” with the plug icon on the left (Figure 3B).
  d. Reconfigure the syringe settings (Figures 3C and 3D).
    i. Select syringe type: Glass, Volume: 1 mL.
    ii. Click “Apply syringe”.
  e. Test the syringe pump (see white box in Figure 3E).
    i. Click the Refill syringe icon and confirm the plunger moves appropriately.
    ii. Click the Stop dosing icon to halt movement.
    iii. Set the syringe level at ∼200 μL to secure its correct position upon mounting.
32. Mount the syringe into the pump and secure it using two fixative bolts (Figure 4A).
  a. Attach the appropriate plunger holder according to the plunger size.
  b. Ensure the syringe is firmly secured and does not move.

**Figure 4:**
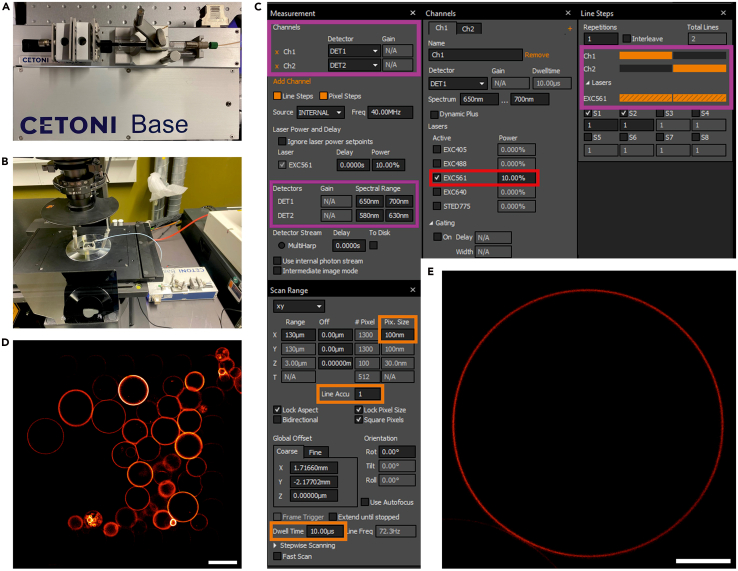
Microfluidic unit assembly, and confocal acquisition of GUV images in the microfluidic trapping device (A) Microfluidic device with syringe connection and fixation on the syringe pump. (B) Overview of the fully assembled microfluidic unit on the microscope stage. (C) Demonstration of GUV confocal scanning parameters on the control panel of the Imspector software. (D) Confocal image of GUVs inside a trap, acquired with an Olympus UPlanXApo 20×/0.8 air objective. Scale bar: 50 μm. (E) Exemplary confocal image of a single L2 GUV acquired using the confocal scanning parameters in (C) at Step 49. Scale bar: 10 μm.
33. Secure the chip along with the syringe on the microscope stage using fixation clamps (Figure 4B).

***Note:*** An overview of the fully assembled microfluidic unit on the microscope stage, connected to the syringe pump, can also be viewed at Figure 4B.

### GUV trapping

**Timing: 1 h**

The following steps describe the procedures for GUV trapping and immobilization via solution exchange and flow rate control. The procedures, detailed for L1 and L2 mixtures at 1 μL/min, is adaptable to different lipid compositions (e.g., softer or stiffer membranes) and alternative flow rates. Effects of different flow rates are highlighted in “limitations”.

34. Configure flow settings in Cetoni software (highlighted with a magenta rectangle in Figure 3E):
  a. Set Volume to −150 μL (negative value indicates pulling).
  b. Set Flow to 1 μL/min.
  c. Click Start icon to begin flow from the inlet reservoir toward the syringe.
  d. Observe fluid movement under Brightfield illumination through the eyepiece and confirm correct flow direction.
35. Set the flow rate to 0 μL/min.
36. Remove the BSA solution from the inlet reservoir using a 200 μL yellow pipette tip.
37. Add 50 μL of S2 to the inlet reservoir.
38. Increase the flow rate to 1 μL/min and allow the solution to fill the chip for 10 min.
39. Reduce the flow rate to 0 μL/min and remove the remaining solution from the inlet reservoir.
40. Add 50 μL of GUV suspension to the inlet reservoir.
41. Increase the flow rate to 1 μL/min and allow GUVs to flow through the chip (Methods video S1).

***Note:*** Aproximate flow duration is 15 min, depending on GUV yield.

42. Use fluorescence microscopy to check whether a sufficient number of GUVs are trapped by the posts in the microfluidic chip.
43. Decrease the flow rate to 0.1 μL/min if enough GUVs are trapped for the experiment.
44. Remove the remaining non-trapped GUV suspension from the inlet reservoir.
45. Add 50–100 μL of S2 to the inlet reservoir.
46. Increase the flow rate to 1 μL/min and allow S2 to fill all traps for at least 30 min.
47. Reduce the flow rate to 0.1 μL/min to apply minimal force to the GUVs.

***Note:*** This flow rate can stabilize GUVs against the posts while maintaining the overall integrity, preparing them for confocal scanning experiments.

Methods video S1. Time-lapse confocal microscopy imaging of the GUV entrapment process, related to step 41 Image sequences were acquired using a 561 nm excitation laser with a 400 μm field of view, and 1 μm pixel size, exported at 10 fps.

### Confocal imaging

**Timing: 2 h**

The following steps describe the setup and imaging procedures for acquiring confocal fluorescence images of trapped GUVs using a commercial Abberior Infinity Line microscope. However, this protocol can be straightforwardly transferred to any other confocal microscope. Imaging parameters should be adjusted as needed based on the available setup in the lab. It is important to note that the confocal imaging for both lipid compositions was performed at 21°C–23°C for consistent GP value comparison. As elevated temperature tends to decrease lipid packing density (lower GP value) and reduced temperature promotes tighter lipid packing (higher GP value).

48. Choose water immersion objective (UPlanSApo 60×/1.2) on the microscope control panel.
49. Turn on the 561 nm excitation laser and allow it to stabilize for at least 30 min before use.
50. Set the laser power to 10 %, corresponding to approximately 5 μW at the sample plane (see rectangle highlighted in red in Figure 4C).

***Note:*** It is recommended to measure the laser power at the sample plane to ensure consistent illumination between different measurements, as the same power percentage may correspond to different absolute laser powers on different setups.

51. Activate two detection channels within the same imaging track as indicated by the line steps, so that signals from both channels are acquired simultaneously after excitation (see rectangles highlighted with magenta in Figure 4C):
  a. Channel 1, emission range: 650–700 nm.
  b. Channel 2, emission range: 580–630 nm.
52. Adjust scan range according to GUV size (maximum scan range in this experimental setup is 133 μm) and set other imaging parameters:
  a. Pixel size: 100 nm.
  b. Pixel dwell time: 10 μs.
  c. Line accumulation: 1 (all details displayed in Figure 4C, rectangles highlighted in orange).

***Note:*** Increase line accumulation (e.g. to 2–4) if the image exhibits low signal-to-noise ratio (SNR). Increasing line accumulation could lead to image artifact demonstrating dislocation of GUV membrane due to sample drift, thus not recommended in general cases. However, in our setup it is possible to increase line accumulation when the native fluorescence signal is low, as the GUVs are immobilized in the chip, longer scanning time and a higher number of scanning lines would not result in distorted GUV membrane shape.

53. Locate the region of interest, use Brightfield mode and eyepieces to locate a trap region with posts.
54. Switch to Fluorescence mode and turn on the 561 nm LED lamp to locate fluorescent GUVs until a trap containing a suitable number of GUVs is observed (Figure 4D).
55. Focus on the equatorial plane of the selected GUV.
56. Acquire an image using the settings above (Figure 4E).
57. Save the image in.msr format. Repeat the above procedures until at least 10 GUV images are collected for each lipid mixture for GP value assessment.

***Note:*** Allow time for the software to complete data acquisition and saving, especially for high-resolution images.

### GP analysis

**Timing: Variable**

The following steps describe how to obtain GP values from the acquired confocal images using a custom macro tool in Fiji/ImageJ, called Fluidity Calculator. This macro tool implements a user-friendly graphical interface for calculating GP values from two-channel data.

**Figure 5:**
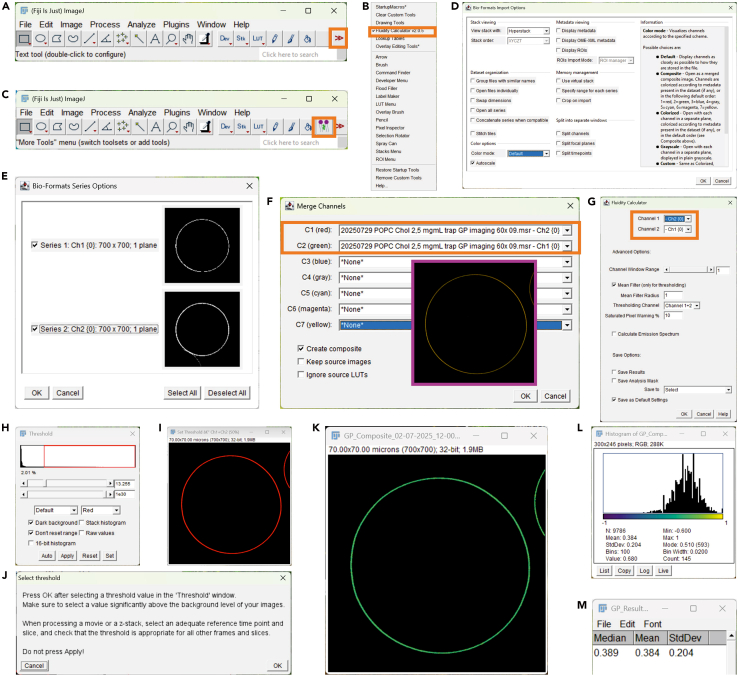
Steps for GP image analysis using the Fluidity Calculator macro in Fiji/ImageJ (A–C) Loading the Fluidity Calculator macro. (D and E) Steps for reading the two-channel Bio-Formats image file. (F) Merging the two channels into a composite image. (G–J) Launching the Fluidity Calculator macro, assigning appropriate channels and intensity threshold. (K–M) Output showing a pseudocolored GP map, GP histogram, and statistical values (mean, median, and standard deviation).
  b. Select Fluidity Calculator from the list (Figure 5B).
  c. The Fluidity Calculator icon will now appear in the toolbar (Figure 5C).

***Note:*** The macro must be loaded each time Fiji is launched.

58. Open Fiji.
59. Load the macro tool.
  a. Click More Tools, see double arrow >> in the Toolbar (Figure 5A).

60. Open an image file either by dragging it into Fiji or using File → Open to locate it, then press OK in the dialog to load the file (Figure 5D).
61. In the Bio-Formats Series Options window, ensure both image series (channels) are selected (Figure 5E).

***Note:*** .msr images are typically imported as two individual grayscale images, each representing one emission channels.

62. Merge the channels into a composite image:
  a. Go to Image → Color → Merge channels.
  b. In Merge Channels window, assign Channel 1 and Channel 2 appropriately (Figure 5F).
  c. Click OK to generate a composite image (Figure 5F, inset highlighted in magenta).
63. Click the Fluidity Calculator icon in the toolbar to launch the macro tool.
64. In the pop-up window, assign channel 1 (*I*_*o*_, ordered channel) and channel 2 (*I*_*d*_, disordered channel), then click OK (Figure 5G).

**CRITICAL:** These channel assignments must remain consistent throughout the analysis.

65. Set an intensity threshold to exclude background noise (Figures 5H and 5I).

***Note:*** The threshold defines which pixels are included in the GP calculation.

66. Press OK to run the analysis (Figure 5J).
67. After processing, several output windows appear:
  a. A pseudocolored GP image map (Figure 5K).
  b. A GP histogram (Figure 5L).
  c. Statistic parameters: mean, median and standard deviation of GP values (Figure 5M).

***Note:*** To ensure consistency and reliable comparisons, maintain the analysis settings, in particular threshold values for all datasets.

## Expected outcomes

**Figure 6:**
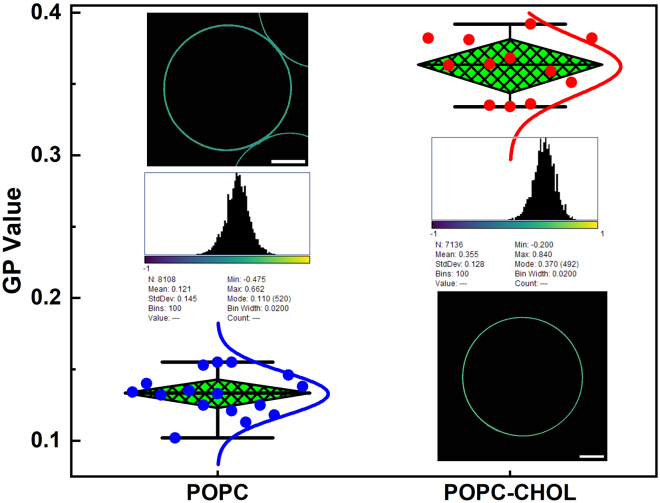
GP analysis of GUVs with different lipid compositions A box plot summarizing the GP values across multiple GUVs, demonstrating a significant difference in membrane order between the two lipid compositions, with blue solid filled circles and Gaussian fit curve indicating GP values for pure POPC (L1), and red solid filled circles and Gaussian fit curve indicating GP values of the mixture for POPC and Cholesterol (L2). Representative GP maps of the GUVs, and the corresponding GP histograms (above or below the box plot) illustrating the distribution of pixel-wise GP values of the GUV membranes are given in each dataset column. Scale bars: 10 μm.

For GP imaging of GUVs, the emission wavelength shifts depending on the membrane lipid order, as sensed by solvatochromic dyes such as NR12A. Higher GP values indicate more ordered (tightly packed) lipid environments, while lower values correspond to more disordered (loosely packed) membrane environments. GP value comparison between the two different lipid compositions is presented in the box plots (Figure 6), with corresponding representative pseudocolored GP images and histograms displayed next to the box plots. L1 GUVs exhibit a mean GP value of 0.1328 ± 0.0152, whereas L2 GUVs have a mean GP value of 0.3623 ± 0.0201. The higher GP value in L2 GUVs reflects the more compact lipid packing induced by cholesterol, which is in line with previous research.^7^

In this protocol, precise GP value determination via confocal scanning is enabled by accurate contour mapping of the GUVs, facilitated by microfluidic manipulation. This approach is suitable for comparative analysis of membrane biophysical properties under varying experimental conditions, such as changes in aqueous environment, lipid composition, and GUV membrane transformations. Consequently, it is particularly valuable for applications in membrane biophysical research, including super-resolution imaging of delicate membrane structures, which often requires prolonged scanning times and stable samples.^13^

## Limitations

When using our microfluidic device, GUVs selected for GP value assessment should not be directly adjacent to the posts. Direct contact with the posts may slightly deform the GUV membrane, potentially influencing GP measurement due to local curvature change. Therefore, it is advised to select GUVs that are trapped adjacent to each other but not in direct contact with the posts.

For GP imaging of GUV membranes, measurements should be performed at the equatorial plane of the GUV. Acquiring GP values away from the equator could result in variations even within the same GUV, leading to unreliable results in comparative analyses.

When labelling GUVs after electroformation, the precise molar ratio of dye to lipid cannot be accurately determined, primarily because the extent of dye incorporation varies among individual vesicles. Consequently, fluorescence signal often differs strongly from one GUV to another (Figure 4D). This heterogeneity complicates quantitative comparisons across vesicles and should be considered when interpreting fluorescence data. As an alternative, the dye can be incorporated into the lipid mixture prior to electroformation, similar to labelling with fluorescent lipid analogues.^1^^,^^5^ This approach generally yields more homogeneous and controlled membrane labelling and eliminates uncertainty in the final dye-to-lipid ratio. However, based on our experience, GUV quality tends to be better when labelling is performed post-electroformation, compared with GUVs prepared directly from pre-labelled lipid and NR12A mixtures. The latter method is still under active investigation, and further optimization and validation are required to fully establish its robustness and reproducibility. Solvatochromic dyes may interfere with GUV formation depending on their charge or hydrophobicity; therefore, it is advised to test a range of dye concentrations (e.g., 0.005–0.5 mol%) to optimize GUV yield and fluorescence intensity without compromising vesicle integrity.

Adjustable experimental parameters include, for example, the flow rate and the GUV production yield. In our experience, trapping efficiency depends more strongly on GUV yield than on the applied flow rate. Provided that the yield is sufficient, most traps can be readily occupied. Although higher flow rates accelerate trap filling, excessively high flow may cause GUVs to adhere firmly to the trapping posts, leading to persistent membrane deformation—particularly in vesicles with low membrane rigidity. Therefore, high flow rates are not recommended. More specifically, the flow rate at which deformation or rupture occurs depends strongly on membrane stiffness and vesicle size. For pure POPC or DOPC GUVs, which exhibit relatively low bending rigidity, slight membrane deformation against the posts was observed even at 1 μL/min during trapping. However, this deformation was reversible upon reducing the flow rate. At 5 μL/min, some larger GUVs displayed pronounced deformation, which became increasingly severe at 10–20 μL/min. GUV rupture was governed by multiple factors, including flow rate, vesicle size, and membrane–post contact area. Larger vesicles tended to rupture more readily than smaller ones as flow rate increases, because greater deformation elevated membrane tension toward the lysis threshold, thereby facilitating irreversible pore nucleation and expansion. For pure POPC GUVs, some smaller vesicles escaped through the post gaps due to substantial deformation, whereas some larger vesicles ruptured on the posts at a flow rate of 50 μL/min. In contrast, GUVs with high cholesterol content (e.g., POPC: CHOL = 40 : 60 mol%) exhibited markedly different behavior. Even at 50 μL/min, these vesicles largely retained a spherical morphology and showed minimal deformation, although strong rotational motion was observed. At 100–200 μL/min, some smaller GUVs were forced through the post gaps without rupturing, while larger vesicles remained intact. Nevertheless, operating at such high flow rates is not advisable. Given that the total internal volume of the microfluidic device is approximately 3 μL, elevated flow rates increase the risk of leakage at connector interfaces and may promote air bubble formation, thereby compromising experimental stability. We therefore recommend operating under mild flow conditions, with an upper practical limit of approximately 10 μL/min. To ensure compatibility with softer lipid compositions and to minimize membrane stress, we demonstrate trapping at 1 μL/min and imaging at 0.05–0.1 μL/min, conditions that provide robust and reproducible results.

## Troubleshooting

### Problem 1

GUVs are not trapped due to their small size (escape through post gaps) (see step 7).

Explanation:

The size of GUVs produced by electroformation is typically heterogeneous, ranging from 1–100 μm. Small vesicles (<10 μm) can flow through the gaps between posts and are therefore not retained within the traps. For optimal trapping efficiency and imaging quality, larger GUVs (approximately 30–60 μm in diameter) are recommended. In this experiment, most of the trapped GUVs were within a range of 20–50 μm.

### Potential solution

Increase the electroformation time and temperature to enhance both the size and yield of GUVs. Additionally, extending the trapping duration may allow a larger number of GUVs from a single batch to be successfully captured.

### Problem 2

Poor visualization of GUVs after electroformation (see step 10).

Explanation:

After GUV collection, it can be challenging to accurately assess their size, quality, and yield using brightfield microscopy. In the original suspension, GUVs tend to move freely and distribute across different focal planes, making quick evaluations difficult. However, assessing these parameters is essential for a smooth start for subsequent microfluidic and imaging experiments.

### Potential solution

Quick observations with brightfield can be effectively achieved by transferring the GUVs (initially in S1) into an osmotically balanced S2 at a 1:10 volume ratio. This creates a mild density gradient that allows GUVs to gradually sediment to the bottom of the observation chamber within a few minutes. The resulting refractive index difference further enhances GUV contrast under transmitted light, enabling clearer visualization after sedimentation.

### Problem 3

Microfluidic chip leakage, or detachment of the PDMS block from the coverslip surface after bonding (see step 19).

### Potential solution

This issue indicates that the covalent bonding between the coverslip glass and PDMS block was incomplete. First, verify that the plasma cleaner is functioning properly by dispensing a tiny drop of water on the coverslip immediately after treatment. If water remains as a droplet on the surface rather than spreading on the surface, the plasma treatment was ineffective and the device may require maintenance. If plasma treatment is confirmed to be effective, inspect the interface between the PDMS block and the coverslip surface for visible air gaps. Gently press the two surfaces together to improve contact if necessary. Finally, consider extending the incubation time for bonding to ensure that the covalent reaction between the PDMS and glass is fully established.

### Problem 4

Trapped GUVs experience oscillatory back-and-forth motion with the flow, caused by pulsation effect from the syringe pump (see step 47).

### Potential solution

For the Nemesys S Syringe pump, the lower operational limit for a 1 mL syringe is 0.0175 μL/min. Operating at or near this minimum flow rate —often done to minimize disturbance to GUVs—can introduce pulsation artifacts that results in reciprocal GUV movement. To ensure stable GUV trapping, it is advisable to avoid the lowest flow settings. We recommend to operate in the range of 0.05–0.1 μL/min, which could facilitate stable vesicle trapping while minimize mechanical disturbance to GUV membrane. Maintaining a smooth and steady imaging process is pivotal for accurately capturing GUV contours and performing reliable lipid packing measurements.

### Problem 5

Fluorescence signals in both channels are saturated (see step 52).

### Potential solution

Reduce the laser power, pixel dwell time, or line accumulations to prevent signal saturation during imaging. Alternately, if the imaging parameters are to be maintained, consider decreasing the dye concentration in the GUVs to decrease overall fluorescence intensity.

## Resource availability

### Lead contact

Further information and requests for resources and reagents should be directed to and will be fulfilled by the lead contact, Ziliang Zhao (ziliang.zhao@leibniz-ipht.de).

### Technical contact

Questions on executing this protocol should be directed to and will be answered by the technical contacts, Gaukhar Zhurgenbayeva (gaukhar.zhurgenbayeva@uni-jena.de) and Ziliang Zhao (ziliang.zhao@leibniz-ipht.de).

### Materials availability

This study did not generate new unique reagents.

### Data and code availability

This study did not generate any codes.

## Acknowledgments

C.E., Z.Z., and G.Z. greatly acknowledge financial support from the 10.13039/501100001659Deutsche Forschungsgemeinschaft (DFG, German Research Foundation; Germany's Excellence Strategy – EXC 2051, project ID 390713860; project no. 316213987 – SFB 1278; GRK M-M-M: GRK 2723/1 – 2023 – ID 44711651; GRK PhInt: GRK 3014/1; instrument funding MINFLUX Jena INST 275_405_1; instrument funding modular STED INST 1757/25-1 FUGG; instrument funding ID 460889961 multi-photon laser scanning device), the State of Thuringia (10.13039/501100010959TMWWDG), the 10.13039/501100001664Leibniz Association (Leibniz ScienceCampus InfectoOptics Jena financed by the funding line Strategic Networking of the Leibniz Association, project no. W8/2018; and Leibniz Collaborative Excellence Programme, project AMPel – project no. K548/2023), the 10.13039/100016019Free State of Thuringia (TAB; AdvancedSTED/FGZ: 2018 FGI 0022; Advanced Flu-Spec/2020 FGZ: FGI 0031; Multi-XUV/2023 FGR 0054), and the innovation program by the German BMWi (ZIM, project 16KN070967/Lab-on-a-chip SMARTIES). Further, this work is supported by the BMFTR (10.13039/501100002347Federal Ministry of Research, Technology and Space) as well as Photonics Research Germany (FKZ: 13N15713/13N15717) and is integrated into the Leibniz Center for Photonics in Infection Research (LPI). The LPI, initiated by Leibniz-IPHT, Leibniz-HKI, UKJ, and FSU Jena, is part of the BMFTR national roadmap for research infrastructures. A part of the project on which these results are based was funded by the 10.13039/100016019Free State of Thuringia under the number 2018 IZN 0002 (Thimedop) and co-financed by funds from the European Union within the framework of the 10.13039/501100008530European Regional Development Fund (EFRE). This research was conducted within the Max Planck School *Matter to Life*, supported by the 10.13039/100031894Dieter Schwarz Foundation in collaboration with the 10.13039/501100004189Max Planck Society. The graphical abstract was created using Biorender.com.

## Author contributions

Conceptualization, Z.Z. and G.Z.; methodology, G.Z., N.O., C.S.M., and Z.Z.; writing, G.Z., N.O., and C.S.M.; review and editing, Z.Z. and C.E.; supervision, C.E. and Z.Z.

## Declaration of interests

The authors declare no competing interests.
